# Evaluation of the Chemical Composition, Bioactive Substance, Gas Production, and Rumen Fermentation Parameters of Four Types of Distiller’s Grains

**DOI:** 10.3390/molecules27186134

**Published:** 2022-09-19

**Authors:** Qi Lu, Qingyuan Luo, Jiaxuan Li, Xu Wang, Chao Ban, Jixiao Qin, Yayuan Tian, Xingzhou Tian, Xiang Chen

**Affiliations:** 1 Key Laboratory of Animal Genetics, Breeding and Reproduction in the Plateau Mountainous Region, Ministry of Education, College of Animal Science, Guizhou University, Guiyang 550025, China; 2 Institute of Animal Nutrition and Feed Science, Guizhou University, Guiyang 550025, China

**Keywords:** distiller’s grains, total phenols, anthocyanin, rumen fermentation, methane

## Abstract

Distiller’s grain is rich in natural active ingredients and can be used as an excellent antioxidant feed for goats. The current study aimed to assess the feeding value of four different types of distiller’s grains with an in vitro gas production trial. The chemical composition, total phenols, total anthocyanins, dry matter degradability, methane, hydrogen, and rumen fermentation parameters were evaluated. The results indicated that red distiller’s grain and glutinous rice distiller’s grain had higher (*p* < 0.05) levels of crude protein than the other two types. There were significantly (*p* < 0.05) higher concentrations of dry matter, ether extract, hemicellulose, and total carbohydrate in corn distiller’s grain than in the other three types of distiller’s grain. In addition, red distiller’s grain showed a higher (*p* < 0.05) gas production rate constant (c) and ruminal outflow rate, as well as higher (*p* < 0.05) concentrations of total phenol, total anthocyanins and 2,2-diphenyl-1-picrylhydrazyl (DPPH) scavenging activity, than the other three types of distiller’s grains. In contrast, red distiller’s grain displayed the lowest (*p* < 0.05) immediately soluble fraction (a) and half the time of maximum gas production relative to the other samples. In particular, the levels of methane (%) in white distiller’s grain and glutinous rice distiller’s grain were greater (*p* < 0.05) than that in red distiller’s grain. Moreover, the ammonia nitrogen content in red distiller’s grain was greater (*p* < 0.05) than that in white distiller’s grain and corn distiller’s grain. In contrast, red distiller’s grain exhibited a lower (*p* < 0.05) level of ruminal fluid acetic acid relative to that found in white distiller’s grain and corn distiller’s grain. Taken together, the results showed that red distiller’s grain and glutinous rice distiller’s grain could be used as protein feed, red distiller’s grain had higher levels of total phenols and total anthocyanins and a high DPPH scavenging activity; corn distiller’s grain might be considered as an alternative energy source feed, and white distiller’s grain exhibited higher total gas production.

## 1. Introduction

Currently, the development of new unconventional functional feed is a research hotspot because the results can provide references and lessons for exploiting and utilizing regeneration feed. Improving the utilization and transformation ratio from grain to livestock products, broadening the available feed resources, and developing unconventional feeds are urgently required [1]. With the increase in global bioethanol production, a large number of byproducts are used for ruminant feedstuffs. Distiller’s grain contains amino acids, vitamins, and trace elements that could be used to produce mixed feedstuffs to realize the development of a circular economy in the liquor-making industry [2]. For instance, Xu et al. [3] demonstrated that distiller’s grain could be used as an alternative fibre source for Holstein calf feed.

The production of methane is the main factor in fermentation energy loss in ruminants, and it undoubtedly contributes to the global greenhouse effect. Reducing methane production is very helpful in improving the energy efficiency of the diet and protecting the environment. Accordingly, it is necessary to find an effective way to decrease methane production in ruminant animals [4]. One study showed that dietary supplementation with natural antioxidants could modulate fermentation parameters, microbial diversity and the microbial metagenome, and may decrease methane production in goats [5]. The inclusion of total phenols in ruminant diets could reduce enteric methane emissions. One study found that a negative correlation was detected between total phenols and methane production [6]. This response occurred because total phenols can affect rumen fermentation, especially the direct inhibition of methanogens and defaunation effects on protozoa, reducing methane emissions. Hence, total phenols are a good predictor of methane reduction potential because they can reduce methane emissions for ruminants [7]. Specifically, total phenols in distiller’s grain may have an antinutritional function, although they can also regulate ruminal fluid fermentation in lactating multiparous Polish Holstein Friesian cows [8]. Moreover, anthocyanins are important bioactive substances in plants, and have many physiological functions and broad prospects for development and application in animals [9]. Notably, distiller’s grain showed a high anthocyanin concentration and a strong antioxidant potential [10]. For example, purple corn anthocyanins could improve antioxidant potential in goat muscle [11].

Distiller’s grain is a byproduct of the liquor industry that is favoured by farmers because of its low production cost and high nutritional value [12]. Previous studies have not acknowledged that distiller’s grain has an abundance of natural active ingredients to improve the health of animals. We hypothesized that distiller’s grain had high total phenol and anthocyanin active ingredients, and could improve antioxidant activity and reduce the methane produced by goats. Therefore, the aim of this study was to evaluate the chemical compositions, total phenols, anthocyanins, biogas production, and rumen fermentation parameters in four types of distiller’s grains by using an in vitro gas production technique.

## 2. Materials and Methods

### 2.1. Materials

The samples consisted of white distiller’s grain (sorghum distiller’s grain; purchased from Guizhou Renhuai Ruida Liquor Co., Ltd., Zunyi China), red distiller’s grain (red yeast rice distiller’s grain; Shengzhou Style Food Co., Ltd., Shaoxing, China), glutinous rice distiller’s grain (Ningde Rongxiang Trade Co., Ltd., Ningde, China), and corn distiller’s grain (the local market in Guiyang). All the samples were dried at 65 °C in a vacuum oven for 72 h and were ground and passed through a 1 mm sieve prior to the experiment for further analysis.

### 2.2. Chemical Analysis

The dry matter (DM), crude protein (CP), ether extract (EE), and ash of distiller’s grains were detected using the method described by the Association of Official Analytical Chemists [13]. Neutral detergent fibre (NDF) and acid detergent fibre (ADF) were analyzed according to the methods of Van Soest et al. [14]. Each sample was run in triplicate. Organic matter (OM) and hemicellulose were calculated using trial and error methods (OM = 100 − ash; and hemicellulose = NDF − ADF). The gross energy was measured using a Parr 6200 calorimeter (Moline, IL, USA). The total carbohydrate (CHO) content was calculated according to the equation adapted from Osborne and Voogt [15]: CHO(%) = 100 − (%moisture + %EE + %CP + %ash).

### 2.3. Bioactive Substances

The total phenolic content of distiller’s grain was analyzed as per Slinkard and Singleton [16]. Briefly, 5 g of distiller’s grain was weighed for total phenol extraction, added to a distilled water volume (solid to liquid) of 1:10, which was acidified with 2 mole of hydrochloric acid to keep the pH < 3, and then added to 0.02 g of acid protease. The supernatant was collected after incubation for 30 min in a 40 °C water bath and centrifuged at 4000× *g* for 10 min at 4 °C (Hunan Kaida Scientific Instrument Co., Ltd., Changsha, China). The sample extract was analysed using the Folin–Ciocalteu method, and the optical density (OD) value was assayed at 725 nm using a microplate reader (Epoch, BioTek, Luzern, Switzerland). Finally, the amount of total phenols was calculated through the standard curve and expressed values on a DM basis (x%).

The total anthocyanin content was determined according to the pH-differential method as described by Yang and Zhai [17]. Briefly, distiller’s grain samples were divided into two portions. One milligram of each sample (two portions) was weighed and placed into a 25 mL volumetric flask. A 25 mL volume of pH 1.0 buffer and pH 4.5 buffer was then transferred into the distiller’s grain sample, and the mixture was shaken vigorously. Absorbance was determined by a microplate reader (Epoch, BioTek, Luzern, Switzerland) at 510 and at 700 nm. The TA content is expressed as cyanidin-3-glucoside equivalents. The total anthocyanin content in distiller’s grain was calculated by the following formula:

total anthocyanin (mg/100 g) = A/EL × 449.2 × D × 25/W × 100,

where A is the absorbance of each sample, E is the cyanidin-3-glucoside molar absorbance (26900), L is the cell path length (1 cm), D is the dilution factor, and W is the dry weight (mg).

### 2.4. DPPH Scavenging Activity

The 2,2-diphenyl-1-picrylhydrazyl (DPPH) scavenging activity was detected according to the method of Tian et al. [18]. Briefly, 0.5 mL of the distiller’s grain extract for the detection of total phenolic compounds was mixed in 1.00 mL of 0.1 mmol/L DPPH (Sigma—Aldrich, Burlington, MA, USA) solution. Next, the culture solution was incubated at 30 °C for 30 min, and the OD was measured at 517 nm by a microplate reader (Epoch, BioTek, Luzern, Switzerland).

### 2.5. In Vitro Gas Production

#### 2.5.1. In Vitro Simulation of Rumen Fermentation Equipment

The design concept, detailed components, and operation methods of anaerobic fermentation equipment in vitro were those used by Wang et al. [19]. Briefly, in vitro anaerobic fermentation equipment consists of a fermentation bottle, constant-temperature incubator, pressure measurement system, gas component detection system, and computer control system. The oscillation frequency in the constant-temperature incubator was 50 r/min and the culture temperature was 39.5 °C. The pressure measurement system comprised a pressure sensor (SMC, Tokyo, Japan) and a three-way solenoid valve (SMC, Japan). The pressure of the fermentation bottle was monitored online through the pressure sensor, and the pressure in the anaerobic bottle was recorded every minute. The three-way solenoid valve opens and releases the pressure of the anaerobic bottle when the pressure in the anaerobic bottle exceeds 9 kPa. Next, the discharged gas enters the gas chromatograph (GC; 7890A, Agilent Technologies Inc., Santa Clara, CA, USA) through the pipe to determine the content of methane and hydrogen. Thus, the production of methane and hydrogen was detected using a GC and was calculated according to the space size at the top of the dispenser (Eppendorf Varispenser, Hamburg, Germany), and the conversion coefficient between pressure and gas volume [20]. The methane for gas production attributes of final asymptotic gas volume (Vf_CH4_) and k were calculated according to Wang et al. [21]; hydrogen as a production attribute of final asymptotic gas volume (Vf_H2_) and k were calculated according to Wang et al. [20].

#### 2.5.2. In Vitro Simulation of Rumen Fermentation Processes

Two Xiangdong black goats equipped with a rumen cannula were used to provide ruminal fluid. The feed was prepared according to National Research Council guidelines [22] with a concentrate/roughage ratio of 40:60. The roughage was rice straw, and the concentrate consisted of (% of DM) 50.00% corn, 27.33% soybean meal, 9.67% wheat bran, 0.33% calcium carbonate, 1.00% monocalcium phosphate, 3.33% oil, 1.67% sodium chloride, and 6.67% premix (1 kg of premix provided 6.9 g of Fe, 4.4 g of Cu, 1.1 g of Co, 11.2 g of I, 11 g of Mn, 4.6 g of Zn, 0.3 g of Se, 104.2 g of Mg, 15.4 g of vitamin mix, and 400 g of sodium bicarbonate). The goats were housed in clean individual pens with free access to water, and the rations offered were 160 g concentrate and 240 g roughage at 8.00 a.m. and 4.00 p.m. Ruminal fluid was collected from three goats before morning feeding through a rumen cannula and was immediately filtered via six layers of absorbent gauze. The ruminal fermentation solution (ruminal fluid:artificial saliva = 1:4) was prepared according to Menke and Steingass [23]. Briefly, 60 mL of ruminal fermentation solution was added to the pipette with 1 g of substrate, and then the fermentation bottle was placed in a constant-temperature incubator, and total gas production was observed. The ruminal outflow rate (k) was calculated according to Wang et al. [21]; the initial fractional rate of degradation (IFRD) and half the time of maximum gas production (HMGP) were also calculated according to Wang et al. [24].

### 2.6. Rumen Fermentation Parameters

The rumen fermentation was stopped at 48 h, and then the pH was immediately determined using a pH meter (FE-28, Mettler Toledo, Switzerland). Next, 2 mL of rumen fermentation solution was centrifuged at 12,000× *g* and 4 °C for 10 min, and then 1 mL of supernatant was mixed with 0.1 mL of 25% meta-phosphoric acid for future analysis of ammonia nitrogen and volatile fatty acids. The remaining samples were filtered through a piece of 300 mesh nylon gauze, placed in an aluminium box, and dried at 105 °C until reaching a constant weight; the dry matter degradability (DMD) was then calculated.

Ammonia nitrogen was assayed according to the method of Weatherburn [25]. Briefly, 10 μL of supernatant was collected into a 1.5 mL tube, added to 300 μL phenol nitrofuran solution and 300 μL base sodium hypochlorite solution, and then shaken vigorously. The OD value was measured at 625 nm by a microplate reader (TACAN, Switzerland) after incubation for 30 min at 37 °C, and ammonia nitrogen content was calculated as per the standard curve.

The volatile fatty acids were analysed via GC, and the peak area of individual volatile fatty acids was detected. We then calculated the concentration according to the standard curve. The GC conditions were as follows: separation of volatile fatty acids was performed using a DB-FFAP column (30 m × 250 μm × 0.25 μm), the carrier gas was nitrogen gas, and the flow rate was 0.8 mL/min; 30 mL of hydrogen and 350 mL of oxygen gas were as combustion gas and 40 mL of N_2_ as eluent for the flame ionization detector; the injection temperature was 250 °C and the split ratio was 50:1, with an injection volume of 1 μL; the temperature program comprised an initial temperature of 60 °C for 2 min, 20 °C/min increase to 220 °C, and maintenance at this temperature for 1 min.

### 2.7. Statistical Analysis

All observations were analysed using SAS System Version 9.1.3 (SAS Institute Inc., Cary, NC, USA). The anaerobic bottle was considered as the experimental unit. There were 3 replicates in each distiller’s grain. Data on chemical composition, gas production kinetics, DMD, and rumen fermentation parameters were analysed by one-way analysis of variance (ANOVA). Data on the total gas production, methane, and hydrogen were noted according to a two-factorial ANOVA model: Y*_ijn_* = μ + S*_i_* + T*_j_* + (S*T)*_ij_* + ε*_ijn_*, where Y*_ijn_* is the observation, μ is the overall mean, S*_i_* is the effect of distiller’s grain, T*_j_* is the effect of incubation time, (S*T)*_ij_* is the effect of interaction between distiller’s grain and incubation time, and ε*_ijn_* is the random error with mean 0 and variance σ^2^. The significant difference level was set at *p* < 0.05.

## 3. Results

### 3.1. Chemical Composition

As shown in Table 1, white distiller’s grain had a significantly higher (*p* < 0.05) level of ADF relative to the other distiller’s grains. Red distiller’s grain showed the highest (*p* < 0.05) OM relative to the other samples. Glutinous rice distiller’s grain had a significantly (*p* < 0.05) higher content of CP, whereas it displayed a lower (*p* < 0.05) CHO content compared to the other three samples. There were significantly (*p* < 0.05) higher concentrations of DM, EE, hemicellulose, and CHO in corn distiller’s grain than in the other three distiller’s grain samples.

### 3.2. Bioactive Substances and DPPH Scavenging Activity

**Figure 1:**
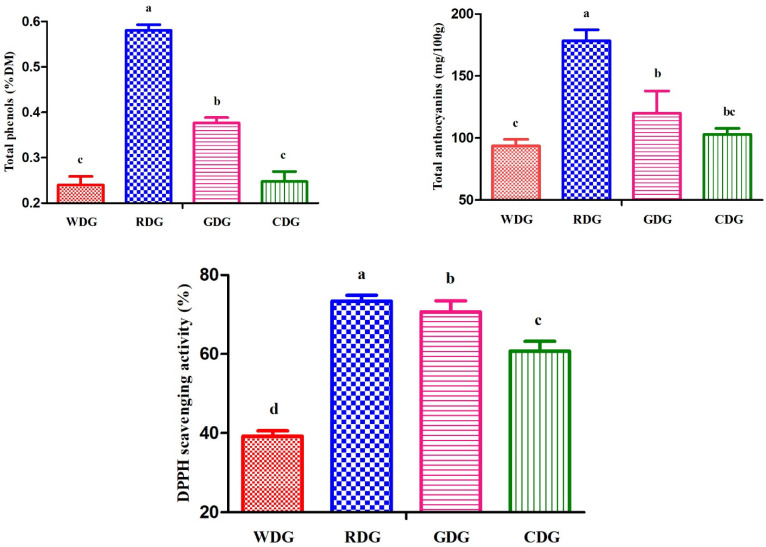
Total phenols, total anthocyanins and DPPH scavenging activity of four types of distiller’s grains. WDG = white distiller’s grains, RDG = red distiller’s grains, GDG = glutinous rice distiller’s grains, CDG = corn distiller’s grains, DPPH = 2,2-diphenyl-1-picrylhydrazyl. Data reported as least-squares means±SD. ^a–d^ Different letters are significantly different (*p* < 0.05).

The levels of bioactive substances for total phenols and total anthocyanins of red distiller’s grain were significantly (*p* < 0.05) greater than those of the other three distiller’s grains (Figure 1). Moreover, the level of total anthocyanins in glutinous rice distiller’s grain was significantly (*p* < 0.05) higher than that in white distiller’s grain, whereas it did not differ significantly (*p* > 0.05) from that found in corn distiller’s grain. Similarly, the DPPH scavenging activity in red distiller’s grain was significantly (*p* < 0.05) higher than that in the other samples.

### 3.3. Total Gas Production Parameters

**Figure 2:**
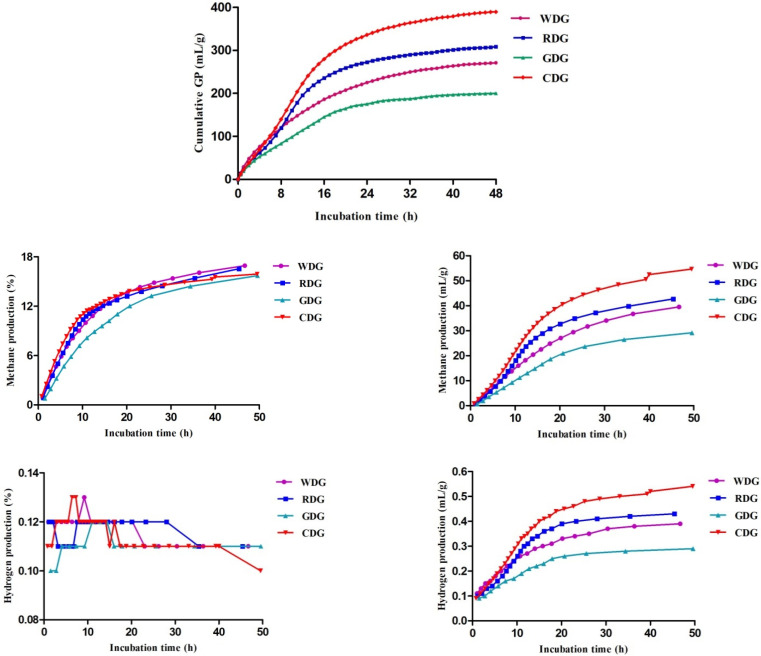
The total gas, methane and hydrogen production of four types of distiller’s grains. WDG = white distiller’s grains, RDG = red distiller’s grains, GDG = glutinous rice distiller’s grains, CDG = corn distiller’s grains. Data reported as least-squares means ± SD.

Corn distiller’s grain showed the highest (*p* < 0.05) total gas production of all the samples (Figure 2). The total gas production was significantly (*p* < 0.05) influenced by the substrate and incubation time. Additionally, white distiller’s grain showed significantly higher (*p* < 0.05) levels of gas production from the immediately soluble fraction (a) and IFRD, whereas it had significantly lower (*p* < 0.05) values of ruminal outflow rate (k) and DMD relative to the other samples (Table 2). Red distiller’s grain had the highest (*p* < 0.05) gas production rate constant (c) and k, as well as the lowest (*p* < 0.05) immediately soluble fraction (a) and HMGP among all the samples. The gas production from the insoluble fraction (b) and potential extent of gas production (a+b) of glutinous rice distiller’s grain were significantly (*p* < 0.05) lower than those of other substrates. Furthermore, the b, a+b, HMGP, and DMD of corn distiller’s grain were greater (*p* < 0.05) than those of white distiller’s grain, red distiller’s grain, and glutinous rice distiller’s grain.

### 3.4. Methane and Hydrogen Production

As shown in Figure 2, the values of methane production tended to increase in response to increasing incubation time for all the distiller’s grains. However, white distiller’s grain showed the highest methane production (%), and corn distiller’s grain displayed higher methane production (mL/g) than the other distiller’s grains. The hydrogen production (%) increased rapidly (*p* < 0.05) up to 10 h of incubation time and then dropped throughout the experiment. In addition, the level of hydrogen production (mL/g) increased (*p* < 0.05) over the whole experimental period, and the hydrogen production (mL/g) in white distiller’s grain was greater (*p* < 0.05) than that of the other samples.

The levels of methane (%) of white distiller’s grain and glutinous rice distiller’s grain did not differ (*p* > 0.05), whereas they were greater (*p* < 0.05) than those of red distiller’s grain and corn distiller’s grain (Table 3). In contrast, red distiller’s grain and corn distiller’s grain showed higher (*p* < 0.05) values of k than the other two samples. Corn distiller’s grain showed the highest (*p* < 0.05) gas volume of methane (V_CH4_) and Vf_CH4_ among all four samples. For hydrogen, no significant difference (*p* > 0.05) was observed in hydrogen (%) among all distiller’s grains. Corn distiller’s grain displayed significantly (*p* < 0.05) higher levels of gas volume of hydrogen (V_H2_) and Vf_H2_ than were found in the other distiller’s grains.

### 3.5. Rumen Fermentation Parameters

Glutinous rice distiller’s grain displayed a higher (*p* < 0.05) pH value relative to the other distiller’s grains (Table 4). The ammonia nitrogen of red distiller’s grain and glutinous rice distiller’s grain was greater (*p* < 0.05) than that of the white distiller’s grain and corn distiller’s grain. White distiller’s grain had a higher (*p* < 0.05) percentage of acetic acid relative to the other distiller’s grains. In contrast, the propionic acid content in corn distiller’s grain was significantly higher (*p* < 0.05) than that in the other distiller’s grains. However, corn distiller’s grain showed a lower (*p* < 0.05) ratio of acetic acid to propionic acid. In addition, glutinous rice distiller’s grain had higher (*p* < 0.05) levels of butyric acid, isobutyrate, valerate, and isovalerate than the other three distiller’s grains.

## 4. Discussion

One previous study revealed that distiller’s grain had an abundance of CP, EE, CF, and minerals and showed high digestibility, making it an ideal nonconventional feed for ruminants [26]. However, there were several reasons for the variation in the chemical composition of distiller’s grain, such as differences in raw materials, processing parameters and methods, concentration of solvent, fermentation yeast, and analysis methods [27]. Mracek [28] proposed that distiller’s grain might vary in terms of chemical composition due to differences in production processes and that the composition could even vary even between different batches made at the same processing plant. As a result, the chemical composition of the four sources of distiller’s grain was varied in the current trial. These observations are in agreement with Böttger and Südekum [29], who indicated that chemical composition was subject to considerable variation among different sources of distiller’s grain. It is worth noting that distiller’s grain is an excellent protein source for ruminants, which can improve ruminant growth performance and reduce the cost of feed [30]. Curzaynz et al. [31] showed that replacing grains and soybean meal with 45% distiller’s grain in the diet of fattening lambs increased their dry matter intake, improved carcass weight, and did not adversely affect carcass characteristics. In the current study, red distiller’s grain and glutinous rice distiller’s grain had relatively high contents of CP (more than 20%) and have potential for use as protein feedstuffs for ruminants.

Polyphenol active compounds are the most abundant natural antioxidant components in plant metabolism for ruminants [32]. Plant polyphenol compounds have anti-inflammatory, antibacterial, antitumour, and antioxidant effects, which might promote the health of the body [33]. Compounds with high levels of polyphenols (including anthocyanins) can eliminate free radicals in the body and regulate the redox state of the organism, improving the antioxidant capacity of the body as well as reducing the risk of cancer, diabetes, and other diseases in animals [34]. Dia et al. [35] reported that an extract of distiller’s grain had 8.15 mg/g total phenols and 1.57 mg/g total anthocyanins. Li et al. [36] showed that white distiller’s grain had 8.81 mg/g total phenols. Lau et al. [37] reported that sorghum distiller’s grain contained 4.63 mg/g total polyphenols. Therefore, the authors assumed that the bioactive substances (such as total phenols and anthocyanins) and superoxide radical scavenging activity and reducing power were improved by distiller’s grain. Moriel et al. [38] showed that wet brewer’s grains contain high antioxidant concentrations, which could enhance the immune function of beef cattle and improve their health condition. Chao [39] revealed that beef cattle receiving distiller’s grain could significantly increase antioxidant activity and reduce the lipid oxidation reaction. Tanaka et al. [40] found that the inclusion of distillers’ dried grains with solubles might be effective in reducing oxidative stress in dairy cows during the hot season because it has the potential to remove excessive free radicals by reducing plasma thiobarbituric acid reactive substances. In the current report, we found that four distiller’s grains showed an abundance of total phenol and anthocyanin concentrations and a high level of DPPH scavenging activity, especially red distiller’s grain, suggesting that it can remove excessive free radicals, improve the antioxidant performance of the body, and protect the health of ruminants.

The rumen microbiota is very important for the digestion of ruminants; these organisms can ferment feed and provide fermentation end products that can be used by host animals. A diet high in CHO would be degraded by microorganisms and produce more gases. Zhao et al. [8] found that CHO in the plant had a higher degradation rate in the rumen because it contained less fibre and is easy to ferment in the ruminal fluid of beef heifers. Thus, corn distiller’s grain exhibited a high level of total gas production, perhaps because it had higher levels of fibre concentration and a high level of CHO, leading to a faster degradation rate. In the current study, red distiller’s grain and corn distiller’s grain showed negative gas production from an immediately soluble fraction of “a”. It is likely that rumen fermentation can be restricted by microbial colonization as well as by some soluble components in red distiller’s grain and corn distiller’s grain that has been consumed, whereas cell wall fermentation had not yet begun [41]. Huang et al. [42] demonstrated that high polyphenol plants could reduce methanogen activities by decreasing hydrogen reduction in the rumen by an in vitro gas production technique. Thus, the corresponding values of IFRD in red distiller’s grain and corn distiller’s grain showed a markedly decreasing trend. In addition, the gas production rate constant of “c” indicates the passage rate of nutrients through the rumen. Our findings revealed that corn distiller’s grain displayed a low level of “c”, which may suggest that the retention time of corn distiller’s grain in the rumen was long, resulting in high DMD; this assumption was also verified in the experiment. Moreover, lignin could reduce the availability and utilization of CHO owing to the cell walls of the plant, making CHO less accessible and more difficult to digest and utilize for ruminants [43].

Wysocka et al. [44] proposed that distiller’s grain could improve rumen fermentation characteristics by increasing total volatile fatty acids and changing the proportion of some acids, resulting in reduced energy losses associated with methanogenesis. Hydrogen is an intermediate product accompanied by volatile fatty acids and can suppress coke formation during methane conversion and also enhance methane conversion. In general, more hydrogen indicated more methane in the ruminant. The polyphenol active component, as a secondary metabolite of plants, is widely involved in methane emission reduction in the rumen [45]. Thus, bioactive substances may stimulate bacterial growth or alter ruminal fluid microbial populations, which could improve feed digestion and utilization in the rumen. For instance, Patra et al. [46] showed that the total phenols in the plant showed inhibitory properties against methanogenesis by decreasing the protozoal populations. Hence, red distiller’s grain contained bioactive substances and showed a lower level of methane (%) in this study. Corn distiller’s grain is rich in EE, which might reduce enteric methane emissions in ruminants [47]. In the present study, corn distiller’s grain showed a low level of methane, possibly related to the high EE content. Furthermore, propionic acid is an end product of the fermentation of various bacterial species in the rumen, and the propionic acid fermentation pathway is distinguished from the pathways resulting in acetic acid and butyric acid not liberating hydrogen [48]. Corn distiller’s grain and red distiller’s grain displayed a high level of propionic acid, which meant that they could stimulate propionic acid fermentation in the ruminal fluid of goats, thus inhibiting methane emission. This was probably due to the ability of the polyphenols to form complexes by bonding to metal ions, amino acids, and polysaccharides [49]. On the one hand, polyphenols (including total phenol and anthocyanin) could promote the flow of microbial protein, improve feed utilization, and reduce methane production in the gastrointestinal tract; on the other hand, polyphenols might also combine with rumen nutrients (such as protein) to form complex nutrients, which may be digested and absorbed through the rumen to the small intestine, improving feed utilization efficiency and reducing methane emissions [50]. This conjecture needs to be determined in further studies. Consistent with our result, Hünerberg et al. [51] found that distiller’s grain could reduce methane emissions from the gastrointestinal tracts of beef cattle. Castillo-Lopez et al. [52] suggested that supplementing corn distiller’s grain in dairy cattle diets may decrease the proportion of dietary energy wasted in the form of methane, increasing energy efficiency. Distiller’s grain is rich in active substances for total phenols and anthocyanins, which can inhibit methane production in the rumen by regulating the rumen fermentation type and methanogenic bacteria [53].

The rumen is a complex microecosystem, and pH is one of the most important factors affecting rumen microorganisms [54]. Corn distiller’s grain showed the lowest pH, perhaps because it contains abundant starch and is easy to degrade in the rumen. The level of ammonia nitrogen could reflect the rate and extent of the degradation of protein and amino acids in the rumen. Red distiller’s grain and glutinous rice distiller’s grain had relatively high CP, displaying a higher level of ammonia nitrogen than the other two distiller’s grains. Moreover, Kraidees [55] found that the suitable ammonia nitrogen level for rumen microbial growth was 10–20 mmol/L. Hence, red distiller’s grain and glutinous rice distiller’s grain might be more appropriate for rumen balance in terms of ammonia nitrogen content. Suarez-Mena et al. [56] demonstrated that the feeding of 14% distiller’s grain could improve rumen fermentation parameters by increasing ammonia nitrogen and reducing the ratio of acetic acid to propionic acid. Furthermore, distiller’s grain might positively affect methanogenesis, ammonia emission, and the volatile fatty acid profile in the rumen [57]. Hydrogen is consumed in propionic acid fermentation to decrease methane production, increasing the energy utilization rate and improving the environment. Moreover, the high carbon loss caused by a high concentration of propionic acid at a high substrate concentration is not conducive to subsequent methane fermentation in ruminants [58]. In the present trial, corn distiller’s grain and red distiller’s grain had a high level of propionic acid, whereas they showed a relatively low ratio of acetic acid to propionic acid, suggesting that the yield of methane is low, and the energy utilization rate of feed is higher. Consistent with our study, Miśta et al. [59] showed that distiller’s grain could reduce the total gas production, total volatile fatty acid, ammonia nitrogen, and methane during in vitro ruminal fluid fermentation.

## 5. Conclusions

The current research indicates that (1) corn distiller’s grain showed a relatively high CHO concentration and might be considered an alternative energy source feed; (2) red distiller’s grain has an abundance of total phenols and total anthocyanins and shows a high level of DPPH scavenging activity, which could reduce methane production; (3) glutinous rice distiller’s grain had a relatively high content of CP and could be used as a protein feedstuff; and (4) white distiller’s grain was superior to other distiller’s grains in terms of improving gas production. Further studies are needed to understand the mechanism of active constituents of active substances in distiller’s grain inhibiting rumen methane with in vivo experiments.

## Figures and Tables

**Table 1:** Chemical composition of four types of distiller’s grains.

Parameter ^1^	WDG ^2^	RDG	GDG	CDG	SEM	*p*-Value
DM (%)	84.16 ^c^	87.01 ^b^	84.28 ^c^	89.24 ^a^	0.3983	<0.0001
Ash (% of DM)	4.77 ^d^	1.12 ^a^	2.63 ^c^	2.18 ^b^	0.0551	<0.0001
OM (% of DM)	95.23 ^d^	98.88 ^a^	97.37 ^c^	97.82 ^b^	0.0551	<0.0001
CP (% of DM)	17.00 ^c^	33.73 ^b^	44.19 ^a^	8.59^d^	0.4729	<0.0001
EE (% of DM)	6.09 ^b^	2.82 ^c^	2.47 ^c^	11.92 ^a^	0.1405	<0.0001
GE (MJ/kg of DM)	18.65 ^c^	21.02 ^b^	21.96 ^a^	18.23^d^	0.0252	<0.0001
NDF (% of DM)	25.81 ^b^	19.21 ^c^	20.46 ^b c^	52.63 ^a^	1.8605	<0.0001
ADF (% of DM)	17.21 ^a^	8.73 ^b^	9.14 ^b^	10.50 ^b^	0.6344	<0.0001
Hemicellulose (% of DM)	8.59 ^b^	10.48 ^b^	11.32 ^b^	42.13 ^a^	1.3550	<0.0001
CHO (% of DM)	56.31 ^b^	49.34 ^c^	34.99^d^	66.56 ^a^	0.7443	<0.0001

^1^ Different letters within a row are significantly different *(p* < 0.05). ^2^ WDG, white distiller’s grains; RDG, red distiller’s grains; GDG, glutinous rice distiller’s grains; CDG, corn distiller’s grains; DM, dry matter; OM, organic matter; CP, crude protein; EE, ether extract; GE, gross energy; NDF, neutral detergent fiber; ADF, acid detergent fiber; CHO, carbohydrate; SEM, the standard error of pooled mean.

**Table 2:** Fermentation kinetics and degradability of four types of distiller’s grains.

Parameter ^1^	WDG ^2^	RDG	GDG	CDG	SEM	*p*-Value
a (mL)	7.28 ^a^	−12.21 ^c^	0.88 ^b^	−16.77 ^d^	0.5258	<0.0001
b (mL)	274.20 ^c^	338.83 ^b^	212.07 ^d^	439.07 ^a^	3.2339	<0.0001
a+b (mL)	281.48 ^c^	326.62 ^b^	212.95 ^d^	422.30 ^a^	3.2856	<0.0001
c (% h)	0.0666 ^b^	0.0709 ^a^	0.0664 ^b^	0.0622 ^c^	0.0008	0.0006
k (/h)	0.0689 ^d^	0.1522 ^a^	0.1003 ^c^	0.1347 ^b^	0.0017	<0.0001
IFRD (/h)	0.0689 ^a^	0.0476 ^c^	0.0611 ^b^	0.0432 ^d^	0.0021	<0.0001
HMGP (h)	10.06 ^b^	9.43 ^c^	9.69 ^b c^	10.50 ^a^	0.1265	0.0017
DMD (%)	47.61 ^d^	63.74 ^b^	60.56 ^c^	68.59 ^a^	0.8497	<0.0001

^1^ Different letters within a row are significantly different (*p* < 0.05). ^2^ WDG, white distiller’s grains; RDG, red distiller’s grains; GDG, glutinous rice distiller’s grains; CDG, corn distiller’s grains; a, gas production from the immediately soluble fraction; b, gas production from the insoluble fraction; a+b, potential extent of gas production; c, gas production rate constant; k, ruminal outflow rate; IFRD, initial fractional rate of degradation; HMGP, half the time of maximum gas production; DMD, dry matter degradability; SEM, the standard error of pooled mean.

**Table 3:** Methane and hydrogen production of four types of distiller’s grains.

Parameter ^1^	WDG ^2^	RDG	GDG	CDG	SEM	*p*-Value
CH_4_						
CH_4_ (%)	16.91 ^a^	15.41 ^b^	16.54 ^a^	15.53 ^b^	0.2595	0.0081
VCH_4_ (mL/g)	39.55 ^c^	42.74 ^b^	28.60 ^d^	52.11 ^a^	0.7776	<0.0001
Vf_CH4_ (mL/g)	40.93 ^b^	40.41 ^b^	28.83 ^c^	50.33 ^a^	0.5758	<0.0001
k (/h)	0.09 ^c^	0.17 ^a^	0.12 ^b^	0.16 ^a^	0.0062	<0.0001
H_2_						
H_2_ (%)	0.11	0.11	0.11	0.10	0.0013	0.1825
V_H2_ (mL/g)	0.39 ^c^	0.43 ^b^	0.29 ^d^	0.52 ^a^	0.0061	<0.0001
Vf_H2_ (mL/g)	0.38 ^c^	0.42 ^b^	0.29 ^d^	0.52 ^a^	0.0057	<0.0001
k (/h)	0.10 ^c^	0.20 ^a^	0.11 ^b c^	0.13 ^b^	0.0068	<0.0001

^1^ Different letters within a row are significantly different (*p* < 0.05). ^2^ WDG, white distiller’s grains; RDG, red distiller’s grains; GDG, glutinous rice distiller’s grains; CDG, corn distiller’s grains; CH_4_, methane; H_2_, hydrogen; V, gas volume; Vf, final asymptotic gas volume; k, ruminal outflow rate; SEM, the standard error of pooled mean.

**Table 4:** Rumen fermentation parameters after 48 h of in vitro ruminal fermentation of four types of distiller’s grains.

Parameter ^1^	WDG ^2^	RDG	GDG	CDG	SEM	*p*-Value
pH	6.60 ^c^	6.71 ^b^	6.97 ^a^	6.13 ^d^	0.0055	<0.0001
NH_3_-N (mmol/L)	8.41 ^b^	14.00 ^a^	14.40 ^a^	9.64 ^b^	0.4970	<0.0001
VFA, % molar						
Acetic acid	67.01 ^a^	62.87 ^c^	60.43 ^d^	65.06 ^b^	0.2870	<0.0001
Propionic acid	22.60 ^b^	22.91 ^ab^	19.09 ^d^	23.07 ^a^	0.1099	<0.0001
Butyric acid	7.22 ^c^	7.64 ^b^	8.18 ^a^	7.15 ^c^	0.0975	0.0003
Isobutyrate	0.79 ^c^	1.60 ^b^	3.24 ^a^	0.88 ^c^	0.0715	<0.0001
Valerate	1.29 ^d^	2.07 ^b^	3.09 ^a^	1.72 ^c^	0.0415	<0.0001
Isovalerate	1.10 ^d^	2.91 ^b^	5.98 ^a^	2.11 ^c^	0.1287	<0.0001
Acetic acid to propionic acid ratio	2.96 ^b^	2.92 ^b^	3.17 ^a^	2.82 ^c^	0.0249	<0.0001

^1^ Different letters within a row are significantly different (*p* < 0.05). ^2^ WDG, white distiller’s grains; RDG, red distiller’s grains; GDG, glutinous rice distiller’s grains; CDG, corn distiller’s grains; NH_3_-N, ammonia nitrogen; VFA, volatile fatty acid; SEM, the standard error of pooled mean.

## Data Availability

Data is contained within the article.
